# Can Face- and Smartphone-Touching Behaviors Be Altered with Personal Hygiene Reminders during the COVID-19 Pandemic Period? An Observational Study

**DOI:** 10.3390/ijerph181910038

**Published:** 2021-09-24

**Authors:** Lok-Yee Joyce Li, Shin-Yi Wang, Jinn-Moon Yang, Chih-Jou Chen, Cheng-Yu Tsai, Lucas Yee-Yan Wu, Cheng-Jung Wu

**Affiliations:** 1 Department of Medicine, Shin Kong Wu-Ho-Su Memorial Hospital, Taipei 111, Taiwan; b101102173@tmu.edu.tw; 2 School of Medicine, College of Medicine, Taipei Medical University, Taipei 110, Taiwan; wulucasyeeyan@gmail.com; 3 Department of Otolaryngology, School of Medicine, College of Medicine, Taipei Medical University, Taipei 110, Taiwan; 4 Department of Otolaryngology, Shuang Ho Hospital, Taipei Medical University, New Taipei 23561, Taiwan; 5 National Taiwan University Hospital, Hsinchu Branch, Hsinchu 300, Taiwan; G80733@hch.gov.tw; 6 Institute of Bioinformatics and Systems Biology, National Yang Ming Chiao Tung University, Hsinchu 30010, Taiwan; moon@faculty.nctu.edu.tw; 7 Department of Biological Science and Technology, National Chiao Tung University, Hsinchu 30010, Taiwan; 8 Master Program in School of Nursing, Taipei Medical University, Taipei 111, Taiwan; m432110018@tmu.edu.tw; 9 Department of Civil and Environmental Engineering, Imperial College London, London SW7 2BT, UK; ct619@ic.ac.uk; 10 Ph.D. Degree Program in Biomedical Science and Engineering, National Yang Ming Chiao Tung University, Hsinchu 30010, Taiwan

**Keywords:** mask, smartphone touching, face touching, COVID-19

## Abstract

As part of the new measures to prevent the spread of the 2019 coronavirus disease (COVID-19), medical students were advised to wear a mask in class and avoid touching their faces. Few studies have analyzed the influence of health education on the frequency of face- and smartphone-touching behaviors during the COVID-19 pandemic. This research compared the frequency of in-class face- and smartphone-touching behaviors of medical students before and after the delivery of personal hygiene education during the COVID-19 pandemic. A behavioral observational study was conducted involving medical students at Taipei Medical University. Eighty medical students were recruited during a lecture on otorhinolaryngology. All medical students were required to wear a mask. Their face- and smartphone-touching behavior was observed by viewing the 4 k resolution video tape recorded in class. The recording lasted for 2 h, comprising 1 h prior to the health educational reminder and 1 h afterwards. The frequencies of hand-to-face contact and hand-to-smartphone contact were analyzed before and after the delivery of health education emphasizing personal hygiene. Comprehensive health education and reminders effectively reduce the rate of face- and smartphone-touching behaviors.

## 1. Introduction

Hand hygiene is greatly emphasized because hands are a proven vector for transmitting nosocomial respiratory infections. Advances in cellular technology have led to the development and popularity of smartphones, which are now a part of our daily lives. Between 2011 and 2018, the community-wide rate of adoption and utilization of cell phones skyrocketed from 10% to 60%, and the upward trend is expected to reach 79% by 2025 [1].

Coronavirus disease 2019 (COVID-19), which is caused by severe acute respiratory syndrome coronavirus 2 (SARS-CoV-2), has spread across the globe and affected over 10.4 million global citizens [2]. The main route of transmission for COVID-19 is through infected respiratory droplets. Droplets are small drops of aqueous bodies that are dispersed from the respiratory tract when we speak, cough, or sneeze [3,4,5]. The virus possesses two characteristics that facilitate its rapid spread, namely direct transmission during the presymptomatic and symptomatic period, and indirect transmission from viral particles deposited on surfaces [6]. Upon touching such contaminated surfaces, the virus can then be transmitted into the human body through the mouth, nose, or eyes [7,8].

As part of the new measures to prevent the spread of COVID-19, Taiwan has implemented strict new mask regulations for eight categories of places, including health care facilities, public transportation facilities, places of consumption, places of learning, sports and exhibition venues, entertainment venues, houses of worship, and offices and business venues.

To face this global challenge, community engagement is fundamental for breaking the transmission chain and preventing further community outbreak. Every member of the society has an obligation to strictly adhere to preventive measures, such as proper hand washing, social distancing, and respiratory hygiene, in order to combat infection [2].

Medical students were advised to wear a mask and avoid touching their faces upon implementation of these restrictions. Messages about mouth and nose touching are already commonplace in hand hygiene promotion programs. Increasing medical students’ awareness of their habituated face- and smartphone-touching behaviors, and improving their understanding of self-inoculation as a route of transmission, contribute to enhancing hand hygiene compliance.

Inexpensive and simple, hand hygiene is an effective and practical preventive method to break the colonization and transmission cycle related to self-inoculation. However, few studies have analyzed the influence of public health educational reminders on the frequency of face- and smartphone-touching behaviors during the COVID-19 pandemic. This research compared the frequency of in-class face- and smartphone-touching behaviors of medical students before and after an educational personal hygiene reminder was delivered during the COVID-19 pandemic.

## 2. Materials and Methods

A behavioral observation study involving medical students was conducted at Taipei Medical University. Eighty medical students were recruited when they attended an otorhinolaryngology lecture. All medical students were required to wear a mask. Their face- and smartphone-touching behaviors were observed through digital videotape recording. The video recording lasted for 2 h, consisting of 1 h prior to health education and 1 h afterwards. The educational reminder emphasizing personal hygiene was delivered 1 h after the lesson began. The frequency of hand-to-face contact and hand-to-smartphone contact was analyzed before and after the delivery of the reminder.

### 2.1. Study Design and Participants

This study is an exploratory before–after experimental design. During the first hour of observation, the students were not notified of the experiment. As the first 1 h session ended, a 10 min personal hygiene reminder, including educating slides and video, was shown to the participants. The students were informed about this experiment at the same time. They independently signed the consent form. Afterwards, we carried out the second hour of observational study.

Ethical approval was obtained from the Institutional Review Board of Taipei Medical University prior to the commencement of the study. Informed consent was obtained from all students. All methods were conducted in accordance with relevant guidelines and regulations.

### 2.2. Data Collection

The in-class face- and smartphone-touching behavior of each student was observed throughout the 120 min lesson and recorded using a digital videotape device. All medical students were required to wear a mask throughout the lecture. The video recording lasted for 2 h, consisting of 1 h prior to the health educational reminder and 1 h afterwards. This health educational reminder, emphasizing personal hygiene, was delivered 1 h after the lesson began. The students were educated about proper hand hygiene and they were instructed not to touch their smartphones or faces to prevent transmission of infectious diseases. The frequency of hand-to-face and hand-to-smartphone contact was analyzed before and after the personal hygiene reminder. The videotape recording was viewed by a researcher (Y.L.A.K.) after the lecture. The researcher used a standardized scoring sheet to record the number of times that each observed person touched their facial zone (unmasked area), masked area, and smartphone.

### 2.3. Statistical Analysis

Descriptive statistics were applied to determine touching frequencies using SPSS version 21 for Windows (SPSS, Chicago, IL, USA). The total numbers of face and smartphone contacts were summed. We compared the behavioral pattern, specifically the frequencies of face- and smartphone-touching behaviors, 1 h before and 1 h after the personal hygiene educational reminder was delivered. The mean and standard deviations were calculated, and a significant difference was tested using a paired *t* test.

## 3. Results

**Figure 1:**
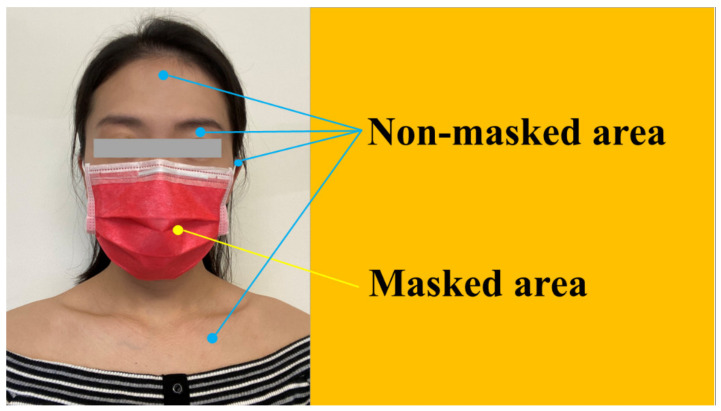
Face-touching behavior during COVID-19 pandemic, depicting masked and unmasked areas.

Before the personal hygiene message was delivered, of the 80 medical students, 73 were observed touching their faces at least once in 1 h and only 7 were not. The total number of face touches of all recruited students reached 777 in 1 h, with an average of 9.71 ± 7.49 touches per hour (Table 1); 37% (291/777) involved contact with the masked area, and 63% (486/777) involved contact with unmasked areas (Figure 1). They touched their masked and unmasked areas 3.64 ± 3.13 and 6.09 ± 5.35 times per hour on average, respectively (Table 2). Among the 80 students, 71 were observed to have touched their smartphones at least once in 1 h and only 9 were not. The total number of smartphone contacts of all recruited students reached 517 in 1 h, with an average of 6.46 ± 6.94 touches per hour (Table 1).

After the personal hygiene message was delivered, among the 80 students, 70 were observed to have touched their faces at least once in 1 h and 10 were not. The total number of face touches of all recruited students reached 441 in 1 h, with an average of 5.51 ± 4.07 touches per hour (Table 1). Of all face contacts, 44% (196/441) involved contact with their mask, and 56% (245/441) involved contact with unmasked areas. They touched their masked and unmasked areas 2.45 ± 3.09 and 3.06 ± 2.64 times per hour on average, respectively (Table 2). Among the 80 students, 67 were observed to have touched their smartphones at least once in 1 h and only 13 were not. The total number of smartphone touches of all recruited students reached 335 in 1 h, with an average of 4.19 ± 3.86 touches per hour (Table 1).

Before the personal hygiene message was delivered, students touched their faces 9.71 ± 7.49 times per hour on average. After the health educational reminder, they touched their faces 5.51 ± 4.07 times per hour on average. The frequency of face-touching behavior was significantly decreased with the promotion of personal hygiene (9.71 ± 7.49 vs. 5.51 ± 4.07 times per hour, *p* < 0.01; Table 1).

Before personal hygiene education was given, students touched their smartphones 6.46 ± 6.94 times per hour on average. After the health instructions were delivered, they touched their smartphones 5.51 ± 4.07 times per hour on average. The frequency of smartphone-touching behavior was significantly reduced as the participants were educated about personal hygiene (6.46 ± 6.94 vs. 4.19 ± 3.86 times per hour, *p* < 0.01; Table 1).

## 4. Discussion

Amid the COVID-19 pandemic, similar to many other countries, Taiwan’s Central Epidemic Command Center urged the public to wear masks in places where social distancing was not feasible. Masks have been reported to be efficient in prohibiting the transmission of diseases, such as COVID-19, by blocking respiratory droplets and direct facial contact [9,10]. However, the exact mechanisms must be further investigated. The World Health Organization issued mask guidelines advising the use of face masks for personal and public protection when physical distancing is not achievable, such as on public transportation, in public venues, and at workplaces [10,11,12,13]. As a result of personal and public hygiene concerns, students were required to wear face masks and not touch their faces during class. Acting as a mechanical barrier, face masks can both directly and indirectly prevent the transmission of COVID-19 by restricting touching of the mucosal areas of the mouth and nose.

According to our results, after the delivery of personal hygiene education, there was a decline in the rate of face-touching behavior among medical students. Face-touching behavior was further classified into contacts with masked and unmasked facial areas. For masked facial areas, prior to and after the delivery of personal hygiene education, the rate of students touching their masked facial area declined following the announcement of the personal hygiene reminder. We then focused on unmasked facial areas; the students touched their unmasked facial areas less frequently after being reminded about proper hand hygiene. After the hand hygiene reminder was delivered, the rate of smartphone-touching behavior decreased accordingly.

Our study has some limitations. In observational studies such as this, it is better to provide an interrater reliability assessment of the coded variables. In order to avoid possible variance, the data were recorded with the use of a standard score sheet. The researcher used a standardized scoring sheet to record the number of times that each observed person touched their facial zone (unmasked area), masked area, and smartphones. The video clip recorded during the 2 h session was reviewed by one researcher (Y.L.A.K.) who solely calculated and double-checked the number of face touches and smartphone touches. Relying on a single reviewer is a limitation of this study.

It should also be acknowledged as a study limitation that the analysis relies on an uncontrolled before–after experimental design; i.e., there is no control group. Moreover, the number of participants involved in our study was relatively small. This is important given that the study, due to the small sample size of only 80 participants, appears underpowered, especially given that the sub-analyses comparing mask touches or face touches, respectively, rely on an even smaller number of participants. This is a limitation of our study. We had hoped to include more medical students in our study, but some of them did not attend the class due to personal factors.

Another limitation of the study was the inevitable negligence of the Hawthorne effect. The Hawthorne effect refers to people’s inclination to modify their behavior in response to their awareness of being observed in an experiment. Although we could not avoid this effect in the current experiment, we can make use of hidden cameras in further experiments so that we can observe the true response of the students.

Another limitation of the study was the inevitable negligence of the time effect. We observed students’ face- and smartphone-touching behaviors immediately after hand hygiene education, but people are likely to forget these instructions after a certain period of time. Changing habitual behaviors is difficult without repeated reminders, and as a result, the rate of face- and smartphone-touching behaviors may remain unchanged in the long term. We aspire to further investigate the change of students’ face- and smartphone-touching behavior after a certain period of time, be it a week or a month.

Our results correlate with those of previous literature in different aspects. Face-touching behavior is one of the least conducted preventive behavioral changes among all personal protective measures taken by Japanese citizens after the COVID-19 outbreak [14]. A previous study performed in Japan showed that the popularity of many personal protective measures, including social distancing measures, carried out by the general public during the COVID-19 pandemic, improved. However, there is room for improvement, especially in terms of avoiding touching the eyes, nose, and mouth [14]. Monitoring these changes may be relevant when considering practical educational activities to raise and promote awareness of and adherence to the preventive measures [14]. A Japanese study showed that although more people practiced personal protective behavior since the early phase of the COVID-19 outbreak, the frequency of face-touching behavior did not decrease accordingly. Our study indicates the importance of personal hygiene education in changing people’s habitual face-touching behavior.

In addition to wearing a face mask, health education about hand hygiene is paramount. The literature has indicated that proper hand hygiene before and after patient contact is imperative in the prevention of infection transmission [15], especially during symptomatic or asymptomatic prodromal stages when patients are shedding infectious materials and frequently touch their mucosal areas [5]. In particular, clinicians caring for infectious pediatric patients with high shedding concentrations may risk acquiring an infection if they have a high level of face- and smartphone-touching behaviors [15].

Another study suggested that SARS-CoV-2 remains infectious for a few days on glass and banknotes and for up to 6 days on glass and stainless steel. People come into regular contact with living microorganisms when they touch these surfaces with their bare hands. Contaminated hands thus become a vector that transmits the bacteria or virus from a frequently touched surface to a person’s mucosal areas through face touching [16,17]. Because smartphone surfaces can be pathogen carriers, people must abandon the habit of smartphone touching paired with face touching to break the transmission chain of infectious disease. Judging from the aforementioned statistics, comprehensive health education can effectively reduce the rate of students’ health risk behaviors, that is, touching their smartphones and then masked and unmasked facial areas without adequate handwashing.

## 5. Conclusions

This analysis determined that comprehensive health education can effectively reduce the rate of students’ health risk behaviors, that is, touching their smartphones and their masked and unmasked facial areas without adequate handwashing.

## Figures and Tables

**Table 1:** Face- and smartphone-touching behavioral analysis before and after the delivery of a personal hygiene reminder during the COVID-19 pandemic.

	Before Personal Hygiene Reminder	After Personal Hygiene Reminder	*p* Value
Total face-touching incidents	777	441	
Average face-touching incidents, mean ± 1 SD (%)	9.71 ± 7.49	5.51 ± 4.07	<0.01
Total smartphone-touching incidents	517	335	
Average smartphone-touching incidents, mean ± 1 SD (%)	6.46 ± 6.94	4.19 ± 3.86	<0.01

**Table 2:** Face-touching behavioral analysis before and after the delivery of a personal hygiene reminder during the COVID-19 pandemic.

	All Facial Areas	Masked Area	Unmasked Area
Face-touching behavioral analysis before the delivery of a personal hygiene reminder			
Total face-touching incidents	777	291	486
Average face-touching incidents, mean ± 1 SD (%)	9.71 ± 7.49	3.64 ± 3.13	6.08 ± 5.35
Face-touching behavioral analysis after the delivery of a personal hygiene reminder			
Total face-touching incidents	441	196	245
Average face-touching incidents, mean ± 1 SD (%)	5.51 ± 4.07	2.45 ± 3.09	3.06 ± 2.64

## Data Availability

The data that support the findings will be available on request from the corresponding author (e-mail: b101090126@tmu.edu.tw).

## References

[1] Olsen M., Campos M., Lohning A., Jones P., Legget J., Bannach-Brown A., McKirdy S., Alghafri R., Tajouri L. (2020). Mobile phones represent a pathway for microbial transmission: A scoping review. Travel Med. Infect. Dis..

[2] World Health Organization Advice on the Use of Masks in the Context of COVID-19. Interim Guidance 6 June 2020. https://www.who.int/publications/i/item/advice-on-the-use-of-masks-in-the-community-during-home-careand-in-healthcare-settings-in-the-context-of-the-novel-coronavirus-(2019-ncov)-outbreak.

[3] Wertheim H.F., Melles D.C., Vos M.C., van Leeuwen W., van Belkum A., Verbrugh H.A., Nouwen J.L. (2005). The role of nasal carriage in *Staphylococcus aureus* infections. Lancet Infect. Dis..

[4] Macias A.E., de la Torre A., Moreno-Espinosa S., Leal P.E., Bourlon M.T., Ruiz-Palacios G.M. (2009). Controlling the novel A (H1N1) influenza virus: Don’t touch your face!. J. Hosp. Infect..

[5] Centers for Disease Control and Prevention Clinical Signs and Symptoms of Influenza: Influenza Prevention & Control Recommendations. www.cdc.gov/flu/professionals/acip/clinical.htm.

[6] Peltola V., Waris M., Osterback R., Susi P., Ruuskanen O., Hyypiä T. (2008). Rhinovirus transmission within families with children: Incidence of symptomatic and asymptomatic infections. J. Infect. Dis..

[7] Peltola V., Waris M., Osterback R., Susi P., Hyypiä T., Ruuskanen O. (2008). Clinical effects of rhinovirus infections. J. Clin. Virol..

[8] Thomas Y., Boquete-Suter P., Koch D., Pittet D., Kaiser L. (2014). Survival of influenza virus on human fingers. Clin. Microbiol. Infect..

[9] Barasheed O., Almasri N., Badahdah A.M., Heron L., Taylor J., McPhee K., Ridda I., Haworth E., Dwyer D.E., Rashid H. (2014). Hajj Research Team. Pilot Randomised Controlled Trial to Test Effectiveness of Facemasks in Preventing Influenza-like Illness Transmission among Australian Hajj Pilgrims in 2011. Infect. Disord. Drug Targets.

[10] Shiraly R., Shayan Z., McLaws M.L. (2020). Face touching in the time of COVID-19 in Shiraz, Iran. Am. J. Infect. Control.

[11] Wang H., Chen M.B., Cui W.Y., Xu H.L., Zheng Q.H. (2020). The efficacy of masks for influenza-like illness in the community: A protocol for systematic review and meta-analysis. Medicine.

[12] Chu D.K., Akl E.A., Duda S., Solo K., Yaacoub S., Schünemann H.J. (2020). COVID-19 Systematic Urgent Review Group Effort (SURGE) study authors. Physical distancing, face masks, and eye protection to prevent person-to-person transmission of SARS-CoV-2 and COVID-19: A systematic review and meta-analysis. Lancet.

[13] MacIntyre C.R., Chughtai A.A. (2020). A rapid systematic review of the efficacy of face masks and respirators against coronaviruses and other respiratory transmissible viruses for the community, healthcare workers and sick patients. Int. J. Nurs. Stud..

[14] Machida M., Nakamura I., Saito R., Nakaya T., Hanibuchi T., Takamiya T., Odagiri Y., Fukushima N., Kikuchi H., Amagasa S. (2020). Changes in implementation of personal protective measures by ordinary Japanese citizens: A longitudinal study from the early phase to the community transmission phase of the COVID-19 outbreak. Int. J. Infect. Dis..

[15] Chen Y.J., Qin G., Chen J., Xu J.L., Feng D.Y., Wu X.Y., Li X. (2020). Comparison of Face-Touching Behaviors Before and During the Coronavirus Disease 2019 Pandemic. JAMA Netw. Open.

[16] Kwok Y.L., Gralton J., McLaws M.L. (2015). Face touching: A frequent habit that has implications for hand hygiene. Am. J. Infect. Control.

[17] Wellenius G.A., Vispute S., Espinosa V., Fabrikant A., Tsai T.C., Hennessy J., Dai A., Williams B., Gadepalli K., Boulanger A. (2021). Impacts of social distancing policies on mobility COVID-19 case growth in the, U.S. Nat. Commun..

